# Token-splitting improves GPT-4.1 performance on plastic surgery exams: implications for AI-Assisted medical education

**DOI:** 10.1080/10872981.2025.2602788

**Published:** 2025-12-12

**Authors:** Yung-Hsu Lei, Chien-Chung Chen, Ching-Ju Shen

**Affiliations:** a Department of Plastic and Reconstructive Surgery, E-Da Hospital, I-Shou University, Kaohsiung, Taiwan; b School of Medicine, College of Medicine, I-Shou University, Kaohsiung, Taiwan; c Department of Obstetrics and Gynecology, Kaohsiung Medical University Hospital, Kaohsiung Medical University, Kaohsiung, Taiwan

**Keywords:** ChatGPT, large language models (LLMs), artificial intelligence (AI), teaching/learning strategies, medical education

## Abstract

Large language models (LLMs), such as ChatGPT, have demonstrated impressive performance on general medical examinations; however, their effectiveness significantly declines in specialized board examinations due to limited domain-specific training data and computational constraints inherent to their self-attention mechanisms. This study investigates a novel token-splitting strategy informed by Cognitive Load Theory (CLT), aimed at overcoming these limitations by optimizing cognitive processing and enhancing knowledge retention in specialized educational contexts. We implemented a token-splitting approach by segmenting Taiwan plastic surgery board examination materials and associated textbook content into cognitively manageable segments ranging from 4,000 to 20,000 tokens. These segmented inputs were provided to GPT-4.1 via its standard ChatGPT web interface. Model performance was rigorously evaluated, comparing accuracy and efficiency across various token lengths and question complexities classified according to Bloom's taxonomy.The GPT-4.1 model utilizing the token-splitting strategy significantly outperformed the baseline (unmodified) model, achieving notably higher accuracy. The optimal segmentation length was determined to be 6,000 tokens, effectively balancing cognitive coherence with information retention and model attention. Errors observed at this optimal length primarily resulted from content absent from textual materials or requiring multimodal interpretation (e.g., image-based reasoning). Provided relevant textual content was adequately segmented, GPT-4.1 consistently demonstrated high accuracy (From 75.88% to 92.93%). The findings highlight that a token-splitting approach, grounded in Cognitive Load Theory, significantly enhances LLM performance on specialized medical board examinations. This accessible, user-friendly strategy provides educators and clinicians with a practical means to improve AI-assisted education outcomes without requiring complex technical skills or infrastructure. Future research and development integrating multimodal capabilities and adaptive segmentation strategies promise to further optimize educational applications and clinical decision-making support.

## Introduction

Large language models (LLMs), such as ChatGPT, have shown impressive capabilities in passing general medical examinations [1]; however, their performance significantly deteriorates when dealing with specialised examinations requiring deep, domain-specific knowledge [2–5]. This limitation is particularly evident in non-English contexts and highly specialised fields, such as plastic surgery [2].

Previous studies have demonstrated limited success in overcoming these domain-specific barriers, primarily due to insufficient targeted training data and intrinsic constraints imposed by the self-attention mechanism of LLMs [6]. One prominent challenge is the model's difficulty in processing extensive clinical information, which is fundamental to effective medical education. According to Cognitive Load Theory (CLT), human learning efficiency significantly declines under excessive cognitive demands and information overload [7]. Analogously, ChatGPT's self-attention mechanism becomes less effective when managing overly lengthy textual inputs, impairing its ability to accurately recall structured clinical knowledge [6]. Additionally, recent research highlights that most users lack sufficient understanding of optimal interactions with LLMs, further limiting their practical utility [8].

Current approaches to address these challenges often involve complex fine-tuning or computationally demanding methods, reducing their practical accessibility for educators and clinicians [9–11]. Thus, there remains an urgent need for straightforward, easily deployable strategies applicable within specialised educational contexts.

To bridge this critical gap, this study proposes a novel token-splitting strategy grounded in CLT principles. We hypothesise that strategically dividing extensive clinical texts into cognitively manageable segments can substantially improve GPT-4.1's comprehension and accuracy in specialised medical board examinations. Specifically, we evaluate whether variations in token-length segmentation affect the model's performance, utilising the Taiwanese Board Certification Examination for Plastic Surgeons and the Textbook of Plastic and Reconstructive Surgery as primary evaluation resources.

Ultimately, this research aims to validate a novel, fine-tuning-free token-splitting strategy as a simple yet highly effective solution, distinctively accessible for educators and clinicians to leverage advanced LLM technologies in medical education and clinical practice.

## Materials and methods

### Research questions

In this study, we address two research questions across two phases:

• **Phase 1**: Can GPT-4.1 effectively solve specialised medical board exam questions, and how does it compare with GPT-4o and GPT-4?
• **Phase 2:** Is there a significant association between different token-splitting strategies and the model's answer accuracy and different levels?

These questions guide the development of our token-splitting approach and systematic evaluation under various conditions.

### Phase 1: model implementation and testing protocol (model with no prior content input)

In phase 1, we utilised the OpenAI API [12] to evaluate three large language models (LLMs): GPT-4o (GPT-4 Omni), GPT-4, and the newly released GPT-4.1. GPT-4.1 represents the latest advancement in GPT architecture, promising improved long-context handling, higher accuracy in specialised domains, and enhanced computational efficiency.

Our test corpus consisted of official Taiwan plastic surgery board exam questions from 2020 to 2022, comprising 466 single-choice and 179 multiple-choice questions. All exam questions were included to faithfully replicate real-world testing conditions, irrespective of the presence of images, tables, or charts. No exclusions were made, even in cases of potential discrepancies in content recognition among the evaluated models.

All three models were evaluated via the same API endpoint, using identical procedures to maintain consistency and enable direct comparisons. All data and code are available in the public GitHub repository(Appendix).

For each research question, GPT-4o, GPT-4.1, and GPT-4 were evaluated using standardised exam questions with identical instructions. To avoid memory contamination between questions, each item was assessed in isolated sessions. Accuracy was computed against official answer keys. Detailed implementation protocols, including automation scripts, are provided in the Appendix.

### Phase 2: evaluation of information input strategies and token-splitting conditions (model with prior content input)

Although GPT-4.1 is theoretically capable of processing up to 1,000,000 tokens within a single context window [12], our practical experience indicated that this limit could not be reliably achieved in actual usage. In preliminary testing, attempts to input excessively long, unsegmented content often resulted in significant performance degradation, including reduced accuracy and incomplete content recognition.

To examine the impact of content segmentation on GPT-4.1's accuracy, we evaluated six input strategies based on varying token lengths based on transformer self-attention constraints and Cognitive Load Theory: (1) no prior content input, (2) 4,000-token segments, (3) 6,000-token segments, (4) 10,000-token segments, (5) 20,000-token segments, and (6) entire chapters without segmentation (“non-split”). All input materials were derived solely from the textbook content, and all input strategies were tested only using textbook-derived practice items(481 single-choice) [13]. All questions were categorised using Bloom's taxonomy [14]. Three authors independently coded all questions based on the cognitive operation required to arrive at the correct answer using information in the stem (Table 1); disagreements were resolved through discussion until consensus. Because the distribution across specific Bloom levels was imbalanced, and consistent with prior work [15–17], we collapsed levels into lower-order (Remember, Understand) and higher-order (Apply, Analyse, Evaluate, Create) categories.

**Table 1.** Six cognitive process categories—Remember, Understanding, Apply, Analyse, Create, and Evaluate—along with their brief definitions.

Type	Definition
Remember	Retrieving relevant knowledge from long-term memory.
Understanding	Determining the meaning of instructional messages, including oral, written, and graphic communication.
Apply	Carrying out or using a procedure in a given situation.
Analyse	Breaking down material into its constituent parts and detecting how the parts relate to 1 another and to an overall structure or purpose.
Create	Propose a new solution by integrating ideas.
Evaluate	Making judgments based on criteria and standards.

To mitigate over-segmentation, we used paragraph-aware splitting: whole paragraphs were appended greedily until adding the next would exceed a target (e.g., 6,000 tokens); the chunk then closed at the prior paragraph boundary and the deferred paragraph began the next chunk. Code is available on GitHub and a short demo video on how to feed the model is available (linked in GitHub).The video also demonstrates how to handle situations in which more than 10 segmented files need to be uploaded by simply feeding them to the model in multiple sequential batches.

In Phase 2, to ensure practical relevance and reproducibility for non-technical users, all tests were conducted using the standard GPT-4.1 web interface rather than the API. Token lengths were calculated using OpenAI' s official tokenizer (detailed in Appendix). The model's response accuracy was compared across the six strategies to determine whether moderate segmentation enhances model comprehension and performance in domain-specific educational contexts.

All Phase 2 experiments were conducted using the ChatGPT Team environment, where each conversation window maintains an isolated context. No memory, global context, or custom instruction features were enabled during any part of the study to prevent unintentional information sharing across study arms.

### Statistical analysis

All statistical analyses were performed in Python 3.12.1; packages are listed in Table 2. Descriptive statistics for each token-splitting condition's accuracy was reported as percentages and elapsed time per question (in seconds) are reported as mean ± standard deviation. Reaction times were compared across conditions using a one-way ANOVA. Overall differences in accuracy across all conditions were evaluated with Cochran's Q test. Pairwise comparisons between token-splitting conditions were conducted using McNemar's exact test, with a Bonferroni correction applied to control for multiple comparisons. Under the 6,000-token setting, we conducted pairwise 2 × 2 comparisons of item accuracy among Bloom levels, using χ² tests with continuity correction when all expected counts were ≥5 and Fisher's exact tests otherwise. *p*-values were two-sided and adjusted for multiple comparisons using Holm's method to control the family-wise error rate (*α* = 0.05). All tests were two-sided, and a corrected *p* < 0.05 was considered statistically significant.

**Table 2.** This table maps each statistical analysis to the software package(s) used.

Analysis	Package(s)
Descriptive statistics	NumPy; pandas
One-way ANOVA	SciPy
Cochran's Q test	statsmodels
McNemar's exact test	statsmodels
Multiple-comparison *p*-value adjustment (Bonferroni, Holm)	statsmodels
Pairwise 2 × 2 comparisons	SciPy

## Result

### Phase 1: can GPT-4.1 effectively solve specialised medical board exam questions, and how does it compare with GPT-4o and GPT-4?

We compared the performance of GPT-4.1, GPT-4o, and GPT-4 on Taiwan's plastic surgery board exam questions. GPT-4.1, achieved the highest overall accuracy, particularly in multiple-choice formats. GPT-4o closely followed, with slightly lower accuracy but significantly faster response times. GPT-4 consistently underperformed both newer models.

While these findings demonstrate continued advancements in large language models, none of the tested versions achieved sufficient **accuracy** to support clinical education independently. This highlights the need for targeted strategies—such as structured content delivery through token-splitting—to optimise model performance in specialised educational contexts. Detailed model comparisons are presented in Tables 3 and 4.

**Table 3.** Comparison of GPT-4o, GPT-4.1, and GPT-4 on single-choice questions.

Single	Model 1	Model 2	Accuracy Comparison (%)	Accuracy *p*-value	Time per question (seconds)	Time *p*-value
	GPT-4o	GPT-4	73 ± 3 vs 63 ± 2	< 0.001	0.53 ± 0.17 vs 0.88 ± 0.21	< 0.001
	GPT-4.1	GPT-4	75 ± 4 vs 63 ± 2	< 0.001	0.699 ± 0.154 vs 0.889 ± 0.203	0.011
	GPT-4.1	GPT-4o	75 ± 4 vs 73 ± 3	0.259	0.699 ± 0.154 vs 0.525 ± 0.162	0.009

This table presents the average accuracy and response time for single-choice questions across GPT-4o, GPT-4.1, and GPT-4. GPT-4.1 demonstrates higher accuracy than both GPT-4o and GPT-4, with a statistically significant improvement over GPT-4. While GPT-4o remains the fastest, GPT-4.1 offers a balance of improved accuracy and moderately reduced latency compared to GPT-4.

**Table 4.** Comparison of GPT-4o, GPT-4.1, and GPT-4 on multiple-choice questions.

Multiple	Model 1	Model 2	Accuracy Comparison (%)	Accuracy *p*-value	Time per question (seconds)	Time *p*-value
	GPT-4o	GPT-4	36 ± 4 vs 28 ± 6	0.001	0.52 ± 0.06 vs 1.31 ± 0.30	< 0.001
	GPT-4.1	GPT-4	46 ± 5 vs 28 ± 6	< 0.001	0.82 ± 0.14 vs 1.31 ± 0.30	< 0.001
	GPT-4.1	GPT-4o	46 ± 5 vs 36 ± 4	< 0.001	0.82 ± 0.14 vs 0.52 ± 0.06	< 0.001

This table summarises the average accuracy and response time for multiple-choice questions across GPT-4o, GPT-4.1, and GPT-4. GPT-4.1 significantly outperforms both GPT-4o and GPT-4 in accuracy, with all comparisons reaching statistical significance. While GPT-4o remains the fastest, GPT-4.1 offers a substantial accuracy gain at the cost of moderately increased response time. GPT-4 lags behind both newer models in both speed and performance.

### Phase 2: is there a significant association between different token-splitting strategies and the model's answer accuracy and different levels?

**Figure 1.**
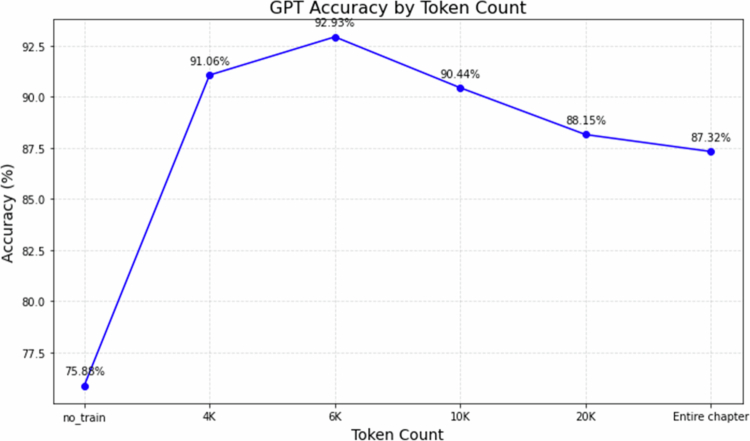
Accuracy Curve Based on Token Count Splits: This figure depicts the accuracy trends observed when data is split based on varying token counts.

As shown in Figure 1, the model without prior content input consistently showed the lowest accuracy. In contrast, the 6,000-token segmentation strategy consistently achieved the highest accuracy across all tested conditions, suggesting it strikes an optimal balance between minimising cognitive load, preserving contextual continuity, and supporting structured knowledge retention.

**Figure 2.**
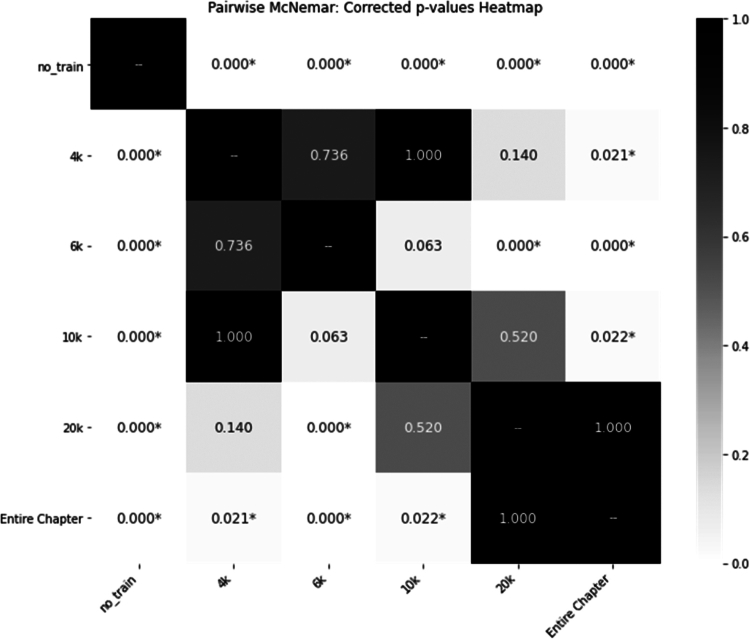
Heatmap of *p*-values for Accuracy Across Token Count Splits: This heatmap visualises the statistical significance (*p*-values) of accuracy differences observed across datasets split by varying token counts.

To further validate these differences, Figure 2 presents a heatmap of *p*-values comparing segmentation strategies. The analysis reveals statistically significant improvements in accuracy for all segmented-input conditions compared to the baseline (*p* < 0.001). Notably, the 6,000-token strategy significantly outperformed both the larger 20,000-token segment and the non-split full-chapter condition (*p* < 0.001), underscoring the advantage of moderate segmentation over both minimal and excessive content chunking.

Exploratory pairwise comparisons among Bloom levels at 6,000 tokens showed no differences after Holm adjustment in Tables 5 and 6.

**Table 5.** Accuracy rates across different strategies for various question types based on Bloom's taxonomy.

Classification	Counts	no train	4k	6k	10k	20k	Entire Chapter
Remember	122	73.77%	92.62%	95.9%	94.26%	92.62%	90.16%
Understanding	122	75.41%	91.8%	90.16%	84.43%	86.07%	86.07%
Analyse	85	72.94%	89.41%	89.41%	87.06%	81.18%	82.35%
Apply	138	79.71%	91.3%	96.38%	95.65%	91.3%	89.86%
Create	0	0%	0%	0%	0%	0%	0%
Evaluate	14	78.57%	78.57%	78.57%	78.57%	78.57%	78.57%

This table illustrates the accuracy rates achieved by different strategies when applied to questions categorised by Bloom's taxonomy.

**Table 6.** Pairwise comparisons of item accuracy between Bloom levels under the 6,000-token split.

Classification1	Classification 2	Number 1	Accuracy 1	Number 2	Accuracy 2	*p*-value
Apply	Evaluate	138	96.38%	14	78.57%	0.267
Evaluate	Remember	14	78.57%	122	95.90%	0.324528
Analyse	Apply	85	89.41%	138	96.38%	0.57703
Apply	Understanding	138	96.38%	122	90.16%	0.57703
Analyse	Remember	85	89.41%	122	95.90%	0.729858
Remember	Understanding	122	95.90%	122	90.16%	0.729858
Evaluate	Understanding	14	78.57%	122	90.16%	0.745817
Analyse	Evaluate	85	89.41%	14	78.57%	1.00
Analyse	Understanding	85	89.41%	122	90.16%	1.00
Apply	Remember	138	96.38%	122	95.90%	1.00

Tests are χ² with continuity correction when all expected counts ≥5, otherwise Fisher's exact; *p*-values are two-sided and Holm-adjusted (Holm–Bonferroni, family-wise α = 0.05).

To address class imbalance, we also report planned collapsed analyses by Bloom level (lower vs. higher order), and Tables 7 and 8 showed no significant accuracy differences. This consistency suggests that token-splitting not only improves LLM accuracy, but also ensures accuracy across a broad range of cognitive processes.

**Table 7.** Accuracy by lower- vs higher-order categories across token-splitting conditions.

Classification	no train	4k	6k	10k	20k	Entire Chapter
Lower-order	74.59%	92.21%	93.03%	89.34%	89.34%	88.11%
Higher-order	77.22%	89.87%	92.83%	91.56%	86.92%	86.5%

This table illustrates the accuracy rates achieved by different strategies when applied to questions categorised by Bloom's taxonomy, highlighting performance variations across cognitive levels such as lower-order (Remember,Understanding) and higher-order (Apply, Analyse and Evaluate).

**Table 8.** Classification results of 6000 token splits.

Classification	Train Size	Accuracy Percentage	*p* value
Lower-order	6,000 tokens	93.03%	
Higher-order	6,000 tokens	92.83%	1.00

This table compares two classification types—lower-order and higher-order—each trained on 6,000 tokens, and provides *p*-values indicating that there is no statistically significant difference in their performance.

## Discussion

Prior work indicates that GPT-4 underperforms in highly specialised fields such as plastic surgery board exams [2]. Consistent with these observations, our results yielded similar accuracy for GPT-4, and performance with later model versions (GPT-4o, GPT-4.1) likewise remained below 80% accuracy in this study. While the medical education community remains optimistic about the future potential of AI-assisted tools, our findings reinforce the reality that frequent inaccuracies cannot be tolerated in clinical training or practice. Despite the rapid evolution of large language models, our results show that the transition from GPT-4o to GPT-4.1 over the past year has not resulted in substantial improvements in domain-specific accuracy. This plateau highlights a critical challenge: effective clinical integration of AI will require novel strategies beyond routine model upgrades.

The persistent underperformance of LLMs in specialised domains appears to stem from two fundamental barriers: a lack of sufficiently targeted training data and inherent computational constraints, particularly the limited capacity of self-attention mechanisms to manage complex, domain-specific information [6]. These limitations mirror the cognitive overload described by Cognitive Load Theory (CLT), where both human learners and AI systems are challenged by excessive information and insufficient structuring. In practice, this often manifests as inconsistent outputs, errors, or even hallucinations when LLMs are confronted with detailed clinical scenarios [18,19]. Overcoming these barriers is essential to unlock the full educational and clinical potential of AI tools, emphasising the urgent need for practical strategies—such as the token-splitting approach explored in this study—to optimise LLM performance in real-world, high-stakes medical education settings.

In response to these challenges, this study introduces a token-splitting strategy specifically designed to address the core limitations of LLMs in specialised clinical assessments, resulting in significant improvements in GPT-4.1's performance on high-stakes examinations. Our approach enables users to enhance model accuracy without the need for complex fine-tuning, and the supporting tools have been made openly available to promote reproducibility and transparency, (see Appendix).

Guided by self-attention constraints and Cognitive Load Theory, we examined segment lengths from 4,000 to 20,000 tokens. As reported in Results (Phase 2), the 6,000-token setting yielded the highest accuracy on our settings. Residual errors with the optimal 6,000-token segmentation were mainly attributable to (1) questions testing knowledge not present in the provided materials and (2) items requiring reasoning beyond the textbook content. Nevertheless, when relevant textual information was available, GPT-4.1 consistently achieved high accuracy using the token-splitting strategy.

The 6,000-token setting reflects two factors. In computer science terms, using a moderate input length reduces attention diffusion in self-attention. Under CLT, it lowers extraneous load while avoiding excessive fragmentation that would hinder cognition. Although 6,000 tokens worked best here, the exact window is task and model dependent. Our recommendation is procedural, not numerical: segment long inputs at a moderate granularity, then tune the segment length on the target task. Thus, we treat segment length as a tunable design parameter rather than a universal constant.

In Phase 2, Bloom-level analyses were intentionally restricted to textbook-derived single-choice items because the Taiwan board items are predominantly “Remember” and highly repetitive across years; including them would distort the cognitive-level distribution. This design choice was planned before analysis and is described in the Methods.

Previous studies [9–11,20] have proposed various backend strategies aimed at reducing computational load and improving the efficiency of LLMs, primarily to lower operational costs. However, these approaches often require advanced technical expertise, thereby limiting their practical usability among general clinical educators and learners. Furthermore, such strategies typically do not effectively address real-world educational challenges, largely due to their limited flexibility in incorporating domain-specific or personalised training materials.

In contrast, our token-splitting approach is designed to be accessible and practical for educators and clinicians. By simply dividing lengthy materials into moderate-sized segments before inputting them into the model, users can substantially reduce information loss and maximise model comprehension. While some minor fragmentation may occur, our findings show that this strategy effectively overcomes the context limitations of current LLMs. As a result, token-splitting offers a straightforward, scalable solution for enhancing LLM performance in specialised medical education contexts, without requiring specialised technical skills.

Beyond its technical simplicity, our approach is also grounded in well-established educational theory. According to Cognitive Load Theory [7], both human learners and computational systems have a limited capacity for processing information. In human learning, large volumes of unstructured content can overwhelm working memory, impeding comprehension [21,22]. Likewise, in LLMs, the self-attention mechanism—which processes and weighs input tokens—faces similar constraints when handling excessively long texts. In large language models, when the input context becomes excessively long, the self-attention mechanism must distribute attention over many tokens, which can dilute task-relevant information and increase the likelihood that earlier content is not effectively used. Conceptually, this reduction in effective use of context is analogous to how extraneous cognitive load interferes with human learning [6]. This perspective is echoed by a recent synthesis integrating CLT with AI, which explicitly recommends simplifying AI inputs and optimising processing to reduce extraneous load, aligning with our paragraph aware, moderate granularity segmentation [23].

Our findings reveal that once ChatGPT is provided with specialised domain knowledge and tested under comparable conditions, its performance significantly improves—regardless of question type. However, effective use of LLMs requires users to understand operational principles and potential pitfalls [8]. This study underscores the ongoing need for clear guidelines, user training, and continuous refinement of LLM-based solutions, particularly in specialised settings. In doing so, we demonstrate that strategic usage and domain-specific preparation can maximise the value of these models.

While the ChatGPT web interface accepts up to 10 files per upload, longer materials can be easily provided in multiple batches. Although we did not perform a controlled comparison of single-batch versus multi-batch uploads, our empirical experience during the study showed no noticeable impact on accuracy or model behaviour. Because the segmented inputs were small plain-text files, file size and upload limits did not impose practical constraints on our token-splitting strategy.

Although this method demonstrated promising results, several limitations should be acknowledged. First, the current implementation exclusively supports segmentation of textual inputs and cannot process image-based or graphical information, restricting multimodal applicability. Second, our evaluation dataset was limited to plastic surgery questions, which may constrain the generalisability of our findings to other medical fields. Finally, our segmentation strategy was tested using token lengths between 4,000 and 20,000 derived from a single textbook source; thus, its effectiveness on larger datasets, diverse textual sources, or multimodal inputs warrants further validation. Further research with larger, more diverse cohorts is necessary to confirm and extend these findings.

### Future directions

In specialised medical education (e.g., plastic surgery), learners require deep domain knowledge and immediate interactive feedback [24], yet persistent gaps between trainee needs and available resources highlight the need for scalable tools [25]. Our study improves specialised medical exam performance in a historically low-baseline field, suggesting broader potential across medical education. Because cognitive overload and information-processing limits recur in complex domains, token-splitting functions as a generalisable instructional design for AI-assisted learning. Given users' limited understanding of effective LLM use [8], educators can pre-segment domain materials and “pre-feed” structured content to models like ChatGPT to reduce cognitive load, and yield more accurate responses. Future work should extend to clinical teaching workflows, including structured lectures, specialty teaching assistants, and standardised patient simulation, while also integrating multimodal inputs and adaptive segmentation, evaluating diverse specialties and non-English contexts, and applying token-splitting to the generation and review of high-stakes exam items [26].

## Conclusion

Token-splitting strategy, informed by CLT, can substantially enhance the performance of LLM in specialised medical education. By segmenting complex domain-specific content, educators and clinicians can significantly improve model accuracy without advanced technical skills or fine-tuning. Our findings highlight the educational value and practical feasibility of this approach for supporting learning and assessment in fields with demanding knowledge requirements. As LLM continue to advance, integrating evidence-based input strategies will be essential to maximise their utility and ensure safe, effective adoption in medical training and clinical practice. Future work should focus on broadening the application of these strategies, incorporating multimodal inputs, and validating their effectiveness across diverse specialties, educational levels, and real-world clinical environments.

## Appendix

We provide two short videos (segmentation workflow; study overview) and the full codebase for Phase 1 (model comparisons) and Phase 2 (Bloom-level analyses and paragraph-aware splitting). We also provided scripts for splitting content into segments. Complete implementation details and code are available in the public repository: [GitHub link: https://github.com/a89182a89182/ChatGPT_Strategies-]. We welcome requests for additional coding resources if readers require them.
